# The association of obstructive sleep apnea with the frailty index in individuals stratified by cardiovascular–kidney–metabolic syndrome stages

**DOI:** 10.1097/MD.0000000000049781

**Published:** 2026-07-10

**Authors:** Can Xing, Xiaoming Guo, Zhenhui Lu, Qin Wang, Yan Li, Xiangyang Zhu

**Affiliations:** a Department of Neurology, Nantong First People’s Hospital Affiliated to Southeast University, Nantong, China.

**Keywords:** cardiovascular, frailty, kidney, metabolic syndrome, obstructive sleep apnea

## Abstract

Association between obstructive sleep apnea (OSA) and frailty progression across various stages of cardiovascular–kidney–metabolic (CKM) syndrome. Data processing and analysis were performed using R version 4.4.0. Multivariate logistic regression models were utilized to assess the association between OSA and Frailty Index. The Cox proportional hazards regression model was utilized to examine the relationship between sleep apnea and frailty across various CKM stages. A total of 5961 participants were included in this study. Compared to the non-frail group (Frailty Index ≤0.21), people with sleep apnea and in the advanced stage of CKM are more prone to developing frailty. In Model 3, adjusted for multiple covariates, the rarely OSA group (odds ratio [OR] = 1.71, 95% confidence interval [CI]: 1.17–2.48), occasionally OSA group (OR = 1.81, 95% CI: 1.25–2.61), and frequently OSA group (OR = 3.08, 95% CI: 1.98–4.79) exhibited an average 71%, 81%, and 208% increase in frailty risk, respectively. For patients with OSA, advanced-stage CKM significantly increases the risk of frailty. Simultaneously, after excluding other confounding factors, patients with advanced-stage CKM had a significantly increased risk of all-cause mortality compared with the population with “no OSA + early-stage CKM.” OSA (especially frequent OSA) and advanced CKM (including progressive stages) are independent and synergistic risk factors for frailty. CKM progression may amplify the adverse effects of OSA on frailty.

## 1. Introduction

Frailty, marked by a reduction in the body’s physiological reserve, disproportionately impacts middle-aged and older populations (particularly those with multiple comorbidities).^[1]^ It is associated with elevated risks of mortality, hospital admission, falls, and placement in long-term care facilities.^[2,3]^ Beyond impairing quality of life, this condition also contributes to increased morbidity and mortality related to cardiovascular disease (CVD).^[4–6]^ Among this vulnerable group, individuals with cardiovascular–kidney–metabolic (CKM) syndrome – a state characterized by the coexistence of multiple chronic conditions – face a heightened risk of frailty progression.^[7,8]^

The concept of CKM syndrome refers to a systemic, progressive health disorder defined by the co-occurrence of obesity, diabetes mellitus, chronic kidney disease (CKD), and CVD.^[9]^ Encompassing cardiovascular, renal, and metabolic dysfunctions, CKM syndrome creates a physiological milieu that promotes frailty development through inflammatory processes, insulin resistance, and disrupted metabolic regulation.^[10]^

The American Heart Association (AHA) Presidential Advisory Statement highlights that sleep disorders, acting as risk-enhancing factors, increase the likelihood of progression across different stages of CKM syndrome.^[9]^ Extensive evidence confirms that unhealthy sleep behaviors represent a significant risk factor for CVD. For instance, snoring has been identified as a factor associated with elevated CVD risk.^[11,12]^ Yang, J. et al further demonstrated that habitual snoring and excessive daytime sleepiness each exhibit an independent association with CVDs in the rural Chinese population.^[11]^

Previous studies have explored the relationship between sleep patterns and frailty.^[13,14]^ Among sleep disorders, obstructive sleep apnea (OSA) is growing in prevalence, with its affected population showing a trend toward younger ages.^[15]^ OSA is a sleep disorder characterized by recurrent breathing pauses during sleep, which result in temporary declines in blood oxygen levels and increases in carbon dioxide levels.^[16]^ These frequent breathing disturbances disrupt the sleep cycle and reduce the duration of deep sleep.

Prior research has established a significant association between OSA and frailty. Yan et al observed a U-shaped relationship between mean pulse oxygen saturation and frailty in patients with OSA; increasing mean pulse oxygen saturation levels may reduce frailty risk and improve prognosis in this population.^[17]^ However, no prior studies have analyzed the association between OSA and frailty advancement across the full spectrum of CKM stages.

This research investigates the association between OSA and frailty progression across various stages of CKM syndrome. Gaining insight into how OSA interacts with different CKM stages to influence frailty risk may provide a foundation for developing early intervention strategies aimed at delaying or preventing the onset of frailty.

## 2. Methods and study population

### 2.1. Study population

This cross-sectional study extracted data from the 2005 to 2008 and 2015 to 2018 cycles of the National Health and Nutrition Examination Survey (NHANES) database. NHANES (a nationally representative survey utilizing stratified, multistage probability sampling) is administered by the National Center for Health Statistics.^[18]^ The reason for selecting data from these 4 cycles is that only these cycles contain data on sleep apnea. This database utilizes a complex probability sampling design that incorporates multistage, stratified, and clustered approaches. More details related to the NHANES database are accessible through the URL: http://www.cdc.gov/nhanes.

**Figure 1.**
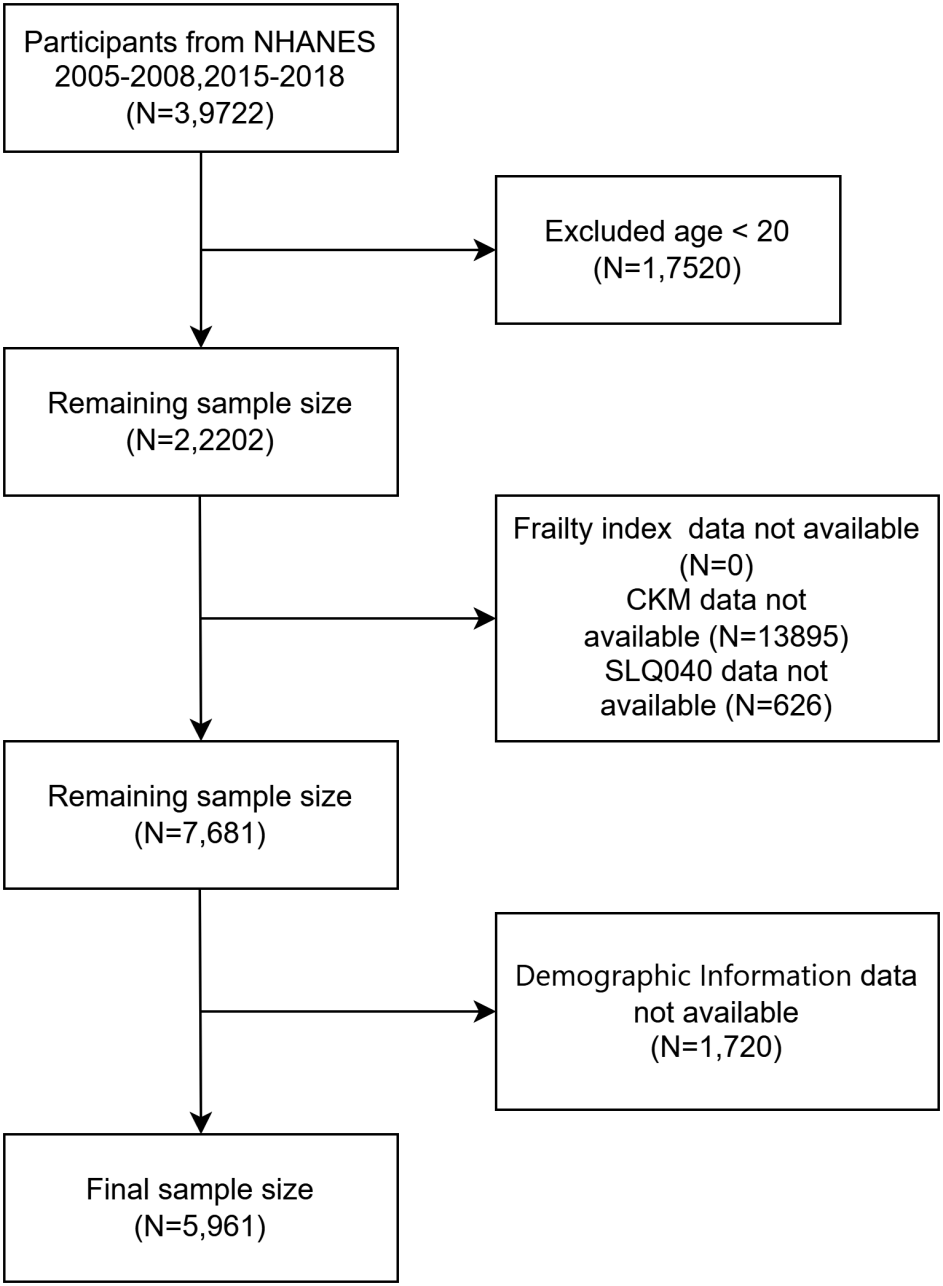
Inclusion flow diagram

In conducting this study, adult participants aged 20 and above who completed both interviews and physical assessments at mobile examination centers were chosen as the research subjects. This study drew on data from 2 time periods: 2005 to 2008 and 2015 to 2018, with a total of 39,722 individuals initially involved. Guided by rigorous inclusion and exclusion criteria, the research team ultimately narrowed down the sample to 5961 U.S. adult participants, selected from the NHANES cycles of 2005 to 2008 and 2015 to 2018. The exclusion process followed clear criteria, removing specific groups of individuals from the initial pool: (1) 17,520 participants who were under 20 years old; (2) 626 individuals with incomplete or missing data related to OSA; (3) CKM syndrome; (4) 1634 individuals with missing values for confounding variables (covariates), which are essential for controlling biases in the analysis (Fig. 1).

### 2.2. Definition of frailty

We developed the frailty index in this study following the standard methodology outlined in references.^[19,20]^ As employed in previous research, the 49-item Frailty Index spans multiple domains such as cognition, dependence, and depression, as well as comorbid conditions, hospital utilization, general health status, anthropometric measurements, and laboratory data.^[19]^ Its development adhered to standardized protocols that had been released earlier.^[20]^ The frailty index is computed as the ratio of the total number of deficits a participant has to the total number of potential deficits. The final value of the frailty index ranges from 0 to 1, where higher values correspond to increased frailty. We further categorized the participants into 2 groups (non-frail and frail), with the classification based on an Frailty Index cutoff of 0.21.^[21]^

### 2.3. Assessment of OSA

Sleep apnea status was reported by participants themselves through a sleep disorder questionnaire, which posed the following question: “During the previous year, how often did you snore, gasp, or have pauses in breathing while asleep?.”^[22]^ Symptoms were categorized according to their occurrence frequency: never, rarely (1–2 nights per week), occasionally (3–4 nights per week), and frequently (5 or more nights per week). Participants who reported experiencing symptoms with a frequency of rarely, occasionally, or frequently were identified as having sleep apnea, and the severity of their sleep apnea was rated based on this frequency. It is important to highlight that the presence and severity of sleep apnea relied exclusively on self-reported symptoms and were not validated via polysomnography.

### 2.4. Assessment of CKM

The AHA has recently defined CKM syndrome, which refers to the interactive relationships between obesity, diabetes, CKD, and CVD.^[9]^ To reflect the progressive characteristics of CKM syndrome, the AHA has further proposed a staging system that covers stages 0 through 4. The stages of CKM syndrome (i.e., stages 0–4) were defined by adapting the criteria from Aggarwal et al to suit the NHANES data.^[23]^ Stage 0 refers to the absence of CKM syndrome risk factors; Stage 1 is defined by a BMI of 25 or above, a waist circumference of 88 cm or more (for females) or 102 cm or more (for males), or prediabetes; Stage 2 included participants with either metabolic risk factors or moderate-to-high-risk CKD, in accordance with the criteria specified in Kidney Disease: Improving Global Outcomes.^[24]^ Stage 3 included participants with very-high-risk CKD (based on Kidney Disease: Improving Global Outcomes criteria) or a high predicted 10-year CVD risk.^[24]^ Additionally, Stage 4 represents established CVD, which includes conditions such as heart attack, coronary heart disease, angina, heart failure, and stroke. Furthermore, participants were grouped into 2 broader CKM syndrome categories: advanced stages (Stage 3 or 4) and early stages (Stage 0, 1, or 2). A high CVD risk was defined as a 10-year risk of 20% or more, calculated using the PREVENT equations.

### 2.5. Measurement of covariates

The covariates included in this study were determined based on both clinical expertise and prior investigations.^[25]^ Age, sex, race, income-to-poverty ratio, drinking status, smoking status, hypertension, diabetes, coronary heart disease, hyperlipidemia, total cholesterol, high-density lipoprotein cholesterol, low-density lipoprotein cholesterol, white blood cell count, lymphocyte percent, monocyte percent, insulin, glucose, and glycohemoglobin were regarded as potential confounding variables. Drinking status was derived from the following question: “In the past 12 months, on those days that {you/SP} drank alcoholic beverages, on the average, how many drinks did {you/he/she} have?.”

### 2.6. Statistical analysis

Weighted *t* tests were used to analyze continuous variables, while weighted chi-square tests were employed to analyze categorical variables. Proportions were used to represent categorical characteristics, while continuous variables were described using means along with their corresponding standard errors.

For the purpose of examining the odds ratio (OR) and 95% confidence interval (CI) between OSA, CKM, and frailty, multiple logistic regression was utilized, with 3 models established: Model 1 adjusted only for age, sex, and race; Model 2 adjusted for age, sex, race, educational attainment, poverty–income ratio (PIR), smoking behavior, and alcohol intake; and Model 3 (fully adjusted), which accounted for age, sex, race, education, PIR, smoking, drinking, hypertension, hyperlipidemia, diabetes mellitus, and CVD. Interaction and subgroup analyses were also carried out to identify possible changes in this association induced by covariates. The Cox proportional hazards regression model was utilized to examine the relationship between sleep apnea and frailty across various CKM stages. Additionally, this model was employed to explore the thresholds of sleep apnea and CKM, along with mortality rates, in patients with frailty.

The threshold for statistical significance was defined as *P* < .05 in this study. Data processing and analysis were performed using R version 4.4.0 (2024-04-24).

## 3. Result

Table 1 presents the baseline features of the study participants. The prevalence of frailty in the adult cohort was 23.5%. All participants had an average age of 49.05 years, and non-Hispanic Whites constituted the majority (68.76%) of the group. Notably, participants with frailty were more likely to be older females with lower education levels, have habits of smoking and drinking, be diagnosed with hypertension, diabetes, hyperlipidemia, CKM, or sleep apnea, and be in the advanced stage of CKM. Additionally, among participants with frailty, levels of glycated hemoglobin, insulin, glucose, and white blood cell count were significantly higher, while levels of low-density lipoprotein cholesterol and lymphocyte percentage were notably lower.

**Table 1:** Baseline characteristics of the participants according to the frailty index.

Variables	Non-frail (n = 4563)	Frail (n = 1398)	*P*
Age, years, mean (SD)	47.11 (0.43)	57.47 (0.53)	<.001
Sex			<.001
Male	2326 (50.30%)	589 (37.70%)	
Female	2237 (49.70%)	809 (62.30%)	
Race			<.001
Mexican American	695 (7.70%)	151 (5.44%)	
Other Hispanic	441 (5.13%)	147 (5.53%)	
Non-Hispanic White	1936 (69.53%)	631 (65.46%)	
Non-Hispanic Black	864 (9.38%)	347 (14.98%)	
Other race	627 (8.26%)	122 (8.59%)	
Education level			**<.001**
<9th grade	375 (4.04%)	197 (8.78%)	
9–11th grade	483 (7.51%)	245 (13.24%)	
High school graduate/GED or equivalent	1012 (22.96%)	369 (30.65%)	
Some college or AA degree	1391 (30.88%)	402 (31.14%)	
College graduate or above	1302 (34.61%)	185 (16.20%)	
Income to poverty ratio	3.28 (0.04%)	2.39 (0.07%)	<.001
Smoking status			<.001
Yes	1885 (42.32%)	793 (58.48%)	
No	2678 (57.68%)	605 (41.52%)	
Drinking status, mean (SD)	2.45 (0.06)	5.12 (2.22)	0.234
Hypertension			<.001
Yes	1442 (28.30%)	974 (67.34%)	
No	3121 (71.70%)	424 (32.66%)	
CVD			<.001
Yes	1559 (32.55%)	778 (55.27%)	
No	3004 (67.45%)	620 (44.73%)	
Diabetes			<.001
Yes	405 (6.34%)	509 (29.84%)	
No	4062 (91.90%)	839 (66.82%)	
Borderline	95 (1.73%)	49 (3.27%)	
Hyperlipidemia			<.001
Yes	1559 (32.55%)	778 (55.27%)	
No	3004 (67.45%)	620 (44.73%)	
Glycohemoglobin (%), mean (SD)	5.54 (0.01)	6.14 (0.04)	**<.001**
Insulin: SI (pmol/L), mean (SD)	72.98 (1.56)	94.55 (2.48)	<.001
Glucose, plasma (mg/dL), mean (SD)	106.54 (0.53)	120.45 (1.07)	<.001
HDL-cholesterol (mg/dL), mean (SD)	55.11 (0.37)	52.93 (0.81)	0.014
LDL-cholesterol (mg/dL), mean (SD)	114.82 (0.76)	108.76 (1.34)	<.001
Total cholesterol (mg/dL), mean (SD)	193.74 (0.93)	191.76 (1.91)	0.306
White blood cell count: SI, mean (SD)	6.69 (0.05)	7.45 (0.08)	<.001
Lymphocyte percent (%), mean (SD)	30.76 (0.18)	29.03 (0.32)	<.001
Monocyte percent (%), mean (SD)	8.22 (0.05)	8.15 (0.10)	0.421
Sleep apnea			<.001
Never	3576 (77.62%)	893 (65.06%)	
Rarely	508 (11.55%)	195 (13.54%)	
Occasionally	272 (6.29%)	146 (9.45%)	
Frequently	207 (4.55%)	164 (11.95%)	
CKM			<.001
0	398 (10.56%)	17 (1.31%)	
1	935 (23.39%)	78 (6.47%)	
2	2759 (57.23%)	711 (53.67%)	
3	201 (4.03%)	106 (7.76%)	
4	270 (4.79%)	486 (30.80%)	
CKM			**<.001**
Early CKM	4092 (91.18%)	806 (61.44%)	
Advanced CKM	471 (8.82%)	592 (38.56%)	

CKM = cardiovascular–kidney–metabolic, CVD = cardiovascular disease.

Logistic regression was employed to explore the relationships between OSA, chronic kidney disease (CKD) stages, and frailty, with the findings summarized in Table 2. When compared to individuals in the “Never sleep apnea” tertile, those categorized in the “Frequently sleep apnea” tertile exhibited a greater likelihood of developing frailty, as indicated by an odds ratio (OR) of 3.13 and a 95% confidence interval (CI) ranging from 2.28 to 4.30. Similarly, participants in the advanced CKD tertile showed a higher tendency toward frailty than their counterparts in the early CKD tertile, with an OR of 6.49 (95% CI: 5.43–7.76).

**Table 2:** Logistic regression analysis on the risk of OSA, CKM, and frailty in NHANES.

Regression model	Crude model OR (95% CI)	*P*	Model 1 OR (95% CI)	*P*	Model 2 OR (95% CI)	*P*	Model 3 OR (95% CI)	*P*
Sleep apnea								
Never	1.00 (Reference)		1.00 (Reference)		1.00 (Reference)		1.00 (Reference)	
Rarely	1.40 (1.05–1.86)	.026	1.50 (1.11–2.02)	.011	1.75 (1.23–2.50)	.003	1.71 (1.17–2.48)	.008
Occasionally	1.79 (1.41–2.28)	<.001	1.81 (1.39–2.36)	<.001	1.81 (1.28–2.57)	.002	1.81 (1.25–2.61)	.003
Frequently	3.13 (2.28–4.30)	<.001	3.35 (2.40–4.68)	<.001	3.70 (2.50–5.47)	<.001	3.08 (1.98–4.79)	<.001
CKM								
Early CKM	1.00 (Reference)		1.00 (Reference)		1.00 (Reference)		1.00 (Reference)	
Advanced CKM	6.49 (5.43–7.76)	<.001	6.02 (4.86–7.46)	<.001	4.81 (3.63–6.36)	<.001	2.27 (1.63–3.15)	<.001

Model 1: Adjusted age, sex, race.

Model 2: Adjusted age, sex, race, education, PIR, smoking, drinking.

Model 3: Adjusted age, sex, race, education, PIR, smoking, drinking, hypertension, hyperlipidemia, diabetes, CVD.

CKM = cardiovascular–kidney–metabolic, CVD = cardiovascular disease, NHANES = National Health and Nutrition Examination Survey, OR = odds ratio, OSA = obstructive sleep apnea.

Even after accounting for the potential confounding effects of age, sex, and race, the connections between OSA, CKD, and frailty remained statistically significant. In Model 3, which incorporated adjustments for all relevant variables, the ORs for frailty across different OSA subgroups were as follows: 1.40 (95% CI: 1.05–1.86) for the “Rarely” subgroup, 1.79 (95% CI: 1.41–2.28) for the “Occasionally” subgroup, and 3.13 (95% CI: 2.28–4.30) for the “Frequently” subgroup. Furthermore, after adjusting for a broader set of factors including age, sex, race, education level, PIR, smoking status, drinking habits, hypertension, hyperlipidemia, diabetes, and CVD, the OR for frailty in participants with advanced CKD (relative to those with early CKD) stood at 2.27 (95% CI: 1.63–3.15).

When examining the dynamic changes in CKM syndrome, patients were split into 2 categories: individuals with persistent early-stage CKM and those with CKM that progressed to the advanced stage. Taking both sleep apnea status and CKM stage into account for subgrouping, the patients were further separated into 4 distinct groups: no sleep apnea/early-stage CKM, no sleep apnea/progressive advanced-stage CKM, presence of sleep apnea/early-stage CKM, and presence of sleep apnea/advanced-stage CKM. Importantly, having sleep apnea alongside advanced-stage CKM was associated with a greater likelihood of elevated frailty risk (Table 3).

**Table 3:** Association between sleep apnea and dynamic changes in CKM stages with frailty.

	Crude model		Adjusted model	
	HR and 95% CI	*P* value	HR and 95% CI	*P* value
No sleep apnea/early CKM stages	1.00 (Reference)		1.00 (Reference)	
No sleep apnea/advanced CKM stages	4.01 (3.46–4.64)	<.001	1.81 (1.45–2.25)	<.001
Presence of sleep apnea/early CKM stages	1.91 (1.58–2.32)	<.001	1.83 (1.51–2.23)	<.001
Presence of sleep apnea/advanced CKM stages	4.86 (3.74–6.32)	<.001	2.14 (1.65–2.78)	<.001

Covariates were described in the part of Section 2.6.

Adjusted model: adjusted age, sex, race, education, PIR, smoking, hypertension, hyperlipidemia, diabetes, CVD.

CI = confidence interval, CKM = cardiovascular–kidney–metabolic, CVD = cardiovascular disease, PIR = poverty–income ratio.

Table 4 presents the results of the subgroup analysis. Findings from this analysis reveal a consistent association between OSA, CKM, and frailty across different subgroups. Notably, no significant interaction effects were observed for sex, race, smoking status, hypertension, educational level, CVD, diabetes, or hyperlipidemia – indicating that the observed association is independent of these variables (all interaction *P*-values > .05).

**Table 4:** Subgroup analyses of the association between obstructive sleep apnea (OSA), cardiovascular–kidney–metabolism syndrome, and frailty across different subgroups.

Variables	OSA	*P* for interaction	CKM	*P* for interaction
	OR (95% CI)		OR (95% CI)	
Sex		.525		.100
Male	1.42 (1.11–1.81)		1.69 (1.36–2.10)	
Female	1.61 (1.18–2.19)		2.28 (1.75–2.97)	
Race		.234		.192
Mexican American	1.66 (0.95–2.91)		1.47 (0.88–2.46)	
Other Hispanic	2.43 (1.47–4.00)		2.56 (1.70–3.85)	
Non-Hispanic White	1.44 (1.10–1.89)		1.69 (1.31–2.18)	
Non-Hispanic Black	2.03 (1.54–2.69)		2.45 (1.85–3.25)	
Other Race	2.21 (1.10–4.44)		2.59 (1.31–5.11)	
Education level		.964		.252
<9th grade	1.57 (0.94–2.62)		2.25 (1.47–3.45)	
9–11th grade	1.41 (0.86–2.30)		1.14 (0.73–1.77)	
High school graduate/GED or equivalent	1.63 (1.03–2.60)		1.93 (1.39–2.69)	
Some college or AA degree	1.64 (1.19–2.25)		2.09 (1.56–2.80)	
College graduate or above	1.40 (0.89–2.23)		1.65 (0.98–2.77)	
Hypertension		.823		.143
Yes	1.26 (1.02–1.56)		1.43 (1.06–1.92)	
No	1.34 (0.87–2.05)		1.95 (1.53–2.49)	
Hyperlipidemia		.070		.249
Yes	1.22 (0.92–1.61)		1.56 (1.21–2.02)	
No	1.81 (1.34–2.46)		1.92 (1.52–2.43)	
Diabetes		.240		.645
Yes	1.27 (0.90–1.79)		1.48 (0.99–2.22)	
No	1.48 (1.18–1.86)		1.81 (1.46–2.25)	
Borderline	3.12 (1.06–9.14)		1.94 (0.82–4.59)	
CVD		.657		.407
Yes	2.72 (0.22–33.08)		1.35 (0.63–2.89)	
No	1.56 (1.25–1.95)		1.86 (1.58–2.20)	
Smoking status		.415		.746
Yes	1.41 (1.07–1.85)		1.83 (1.44–2.31)	
No	1.69 (1.24–2.28)		1.72 (1.33–2.24)	

CI = confidence interval, CKM = cardiovascular–kidney–metabolic, OR = odds ratio.

After adjusting for multiple covariates, both the “no sleep apnea/advanced CKM stages” subgroup (OR = 1.95, 95% CI: 1.37–2.79) and the “presence of sleep apnea/advanced CKM stages” subgroup (OR = 1.72, 95% CI: 1.12–2.65) were significantly associated with all-cause mortality. Their respective risks were 1.95-fold and 1.72-fold higher than that of the “no sleep apnea/early CKM stages” subgroup (Table 5).

**Table 5:** Exploring sleep apnea and CKM thresholds and mortality rates in frailty patients.

Mortality risk	Crude model HR (95% CI)	*P*	Model 1 hour (95% CI)	*P*	Model 2 hours (95% CI)	*P*	Model 3 HR (95% CI)	*P*
All-cause mortality								
No sleep apnea/early CKM stages	1.00 (Reference)		1.00 (Reference)		1.00 (Reference)		1.00 (Reference)	
No sleep apnea/advanced CKM stages	4.87 (3.90–6.08)	<.001	2.12 (1.66–2.72)	<.001	2.30 (1.69–3.13)	<.001	1.95 (1.37–2.79)	<.001
Presence of sleep apnea/early CKM stages	1.17 (0.89–1.54)	0.250	1.23 (0.95–1.59)	0.114	1.45 (0.91–2.28)	0.114	1.36 (0.84–2.20)	0.208
Presence of sleep apnea/advanced CKM stages	3.94 (2.95–5.26)	<.001	2.50 (1.88–3.33)	<.001	2.16 (1.48–3.15)	<.001	1.72 (1.12–2.65)	0.013

Model 1: Adjusted age, sex, race.

Model 2: Adjusted age, sex, race, education, PIR, smoking, drinking.

Model 3: Adjusted age, sex, race, education, PIR, smoking, drinking, hypertension, hyperlipidemia, diabetes, CVD.

CI = confidence interval, CVD = cardiovascular disease, CKM = cardiovascular–kidney–metabolic, PIR = poverty–income ratio.

## 4. Discussion

Frailty, a multidimensional geriatric syndrome characterized by reduced physiological reserve and increased vulnerability to adverse health outcomes, has become a major public health concern amid global population aging. Identifying modifiable risk factors for frailty and its progression is critical for developing targeted prevention strategies. This study systematically explored the associations of OSA, CKM syndrome (including its dynamic progression), and their combinations with frailty and all-cause mortality, yielding several key findings that align with, extend, and innovate upon existing literature.

This study confirmed a strong, dose-dependent association between OSA frequency and frailty risk. Compared with participants with “never sleep apnea,” those with “frequently sleep apnea” had a 3.13-fold higher odds of frailty (95% CI: 2.28–4.30), and this association remained significant even after adjusting for demographic factors (age, sex, race) and comprehensive confounders (lifestyle, comorbidities). Notably, the “rarely” and “occasionally” OSA subgroups also showed elevated frailty risk (OR = 1.40 and 1.79, respectively), indicating a gradual increase in frailty risk with increasing OSA severity.

This finding is consistent with prior epidemiological evidence. In a study conducted by and his research team, it was verified that participants with intermediate or high risk of OSA were more prone to frailty than those with low OSA risk. Specifically, the adjusted odds ratios (along with their corresponding 95% CIs) were 1.67 (1.08–2.56) for the intermediate-risk group and 3.00 (1.69–5.32) for the high-risk group, respectively.^[26]^ The mechanism that drives this association is thought to stem from OSA-related pathological processes: chronic sleep fragmentation, intermittent hypoxemia, and systemic inflammation. These 3 factors collectively compromise muscle function, disrupt energy metabolism, and diminish cognitive reserve (all of which are crucial elements that help maintain resilience against frailty).^[27,28]^ Our study extends this work by stratifying OSA into 4 severity tiers (never/rarely/occasionally/frequently) and confirming a clear dose–response pattern, which strengthens the causal plausibility of the OSA–frailty link.

This study also highlighted the critical role of CKM in frailty, with 2 novel insights. First, advanced CKM was strongly associated with frailty (OR = 6.49 vs early CKD, 95% CI: 5.43–7.76), and this association persisted after adjusting for covariates (OR = 2.27, 95% CI: 1.63–3.15). The pathophysiological processes underlying CKM are characterized by an intricate array of interactive mechanisms, among which oxidative stress serves as a pivotal mediator. On one side, both hyperglycemic conditions and compromised cardiac and renal function can trigger the activation of the renin–angiotensin–aldosterone system. On the other side, these factors also induce elevations in reactive oxygen species, advanced glycation end products, and protein kinase C levels. Collectively, these changes amplify oxidative stress, which in turn further aggravates organ injury and functional impairment.^[29–31]^

Second, we further explored CKM dynamic progression (persistent early-stage vs progressive advanced-stage) and its interaction with OSA. The combination of “sleep apnea + advanced-stage CKM” (either progressive or persistent) was associated with elevated frailty risk, a finding rarely reported in previous literature. This suggests that CKM progression may amplify the adverse effects of OSA on frailty: for example, CKM-induced metabolic dysfunction (e.g., insulin resistance, dyslipidemia) and OSA-related hypoxemia may synergistically exacerbate oxidative stress and pro-inflammatory cytokine release (e.g., TNF-α, IL-6), accelerating the loss of physiological reserve. The findings of these studies emphasize that OSA contributes to the pathophysiological processes underlying CVD, CKD, and metabolic syndrome, and these 3 conditions can induce frailty through mechanisms like inflammation, insulin resistance, and metabolic dysregulation.^[10]^

Subgroup analysis (Table 4) revealed no significant interaction effects of sex, race, smoking status, or comorbidities (hypertension, diabetes, CVD) on the OSA–CKM–frailty associations (all interaction *P* > .05). This indicates that the links between OSA, advanced CKM, and frailty are consistent across diverse populations (whether male or female, White or nonwhite, or with or without traditional cardiovascular risk factors). This robustness is clinically meaningful: it suggests that screening for OSA and monitoring CKM progression may be beneficial for frailty prevention in a broad range of individuals, not just high-risk subgroups. For example, even in nonsmoking or normotensive CKM patients, OSA screening could help identify those at elevated frailty risk.

Beyond frailty, this study linked OSA–CKM combinations to all-cause mortality. After covariate adjustment, participants with “no sleep apnea/advanced CKM” and “presence of sleep apnea/advanced CKM” had 1.95-fold and 1.72-fold higher mortality risk, respectively, compared with “no sleep apnea/early CKM.” This extends the clinical relevance of our findings: OSA and advanced CKM are not only associated with frailty (a “pre-failure” state) but also with a tangible increase in long-term mortality. The mortality association may be mediated by frailty itself—frailty is a well-established predictor of mortality (or by direct pathways [e.g., OSA-related cardiovascular events, CKM-related end-stage renal disease]). Regardless, this finding underscores the urgency of addressing OSA and CKM in clinical practice to improve both frailty outcomes and survival.

The associations observed in this study may be driven by overlapping pathophysiological pathways linking OSA and CKM to frailty: chronic inflammation and oxidative stress: OSA-induced intermittent hypoxemia triggers the release of pro-inflammatory cytokines (IL-6, CRP) and reactive oxygen species; CKM, meanwhile, promotes systemic inflammation via uremic toxin accumulation and impaired renal clearance of inflammatory mediators. Together, these processes accelerate muscle catabolism, reduce mitochondrial function, and weaken immune responses (key contributors to frailty).^[32,33]^ Sleep disruption and metabolic dysregulation: OSA disrupts sleep architecture (e.g., reduced slow-wave sleep), impairing glucose metabolism and insulin sensitivity. CKM exacerbates this by inducing insulin resistance and dyslipidemia. The combination of poor sleep and metabolic dysfunction reduces energy availability for physical activity, further worsening frailty-related physical decline.^[34,35]^ Muscle dysfunction: OSA-related hypoxemia reduces muscle oxygen delivery and impairs protein synthesis; CKM-related hyperparathyroidism and vitamin D deficiency, in turn, promote muscle wasting (sarcopenia). Since sarcopenia is a core component of frailty, this dual hit may explain the synergistic effect of OSA and advanced CKM on frailty risk.^[36,37]^

## 5. Study strengths and limitations

### 5.1. Study strengths

Our findings have important clinical and public health implications. Clinicians should consider screening for OSA in patients with CKM, especially those with progressive advanced-stage CKM. Simple tools (e.g., the STOP-BANG questionnaire) could be used to identify high-risk individuals, who may benefit from further evaluation (e.g., polysomnography). For patients with “OSA + advanced CKM,” interventions targeting OSA (e.g., continuous positive airway pressure therapy) or CKM progression (e.g., renin–angiotensin–aldosterone system inhibitors, glycemic control) may reduce frailty risk. Future interventional studies are needed to test this hypothesis.Frailty prevention in CKM patients should not focus solely on kidney function but also on comorbid conditions like OSA. Integrating sleep health assessments into CKM care plans could improve long-term outcomes, including reduced mortality.

### 5.2. Limitations

Despite its strengths, this study has several limitations. First, as an observational study, it cannot establish causal relationships; residual confounding (e.g., unmeasured factors like physical activity, dietary patterns, or medication use) may have influenced results. Second, the cohort was predominantly non-Hispanic White (68.76%), limiting generalizability to other ethnic groups (e.g., African Americans, Hispanics) who may have higher rates of CKM and OSA. Third, OSA and CKM were not assessed via gold-standard methods (e.g., polysomnography for OSA, repeated eGFR measurements for CKM progression), potentially introducing measurement bias. Finally, frailty may change dynamically, driven by a set of complex and variable factors. Although we have taken steps to adjust for a large number of variables, limitations might still not be fully addressed. Frailty was not assessed longitudinally, so we could not explore the direction of causality (e.g., whether frailty precedes OSA/CKM progression).

## 6. Conclusion

This study demonstrates that OSA (especially frequent OSA) and advanced CKM (including progressive stages) are independent and synergistic risk factors for frailty, with consistent associations across diverse subgroups and links to increased all-cause mortality. These findings highlight the need for integrated management of OSA and CKM to prevent frailty and improve long-term survival. Further research is needed to validate these associations in interventional settings and clarify underlying mechanisms.

## Author contributions

**Data curation:** Xiaoming Guo.

**Funding acquisition:** Xiangyang Zhu.

**Formal analysis:** Can Xing.

**Investigation:** Can Xing.

**Methodology:** Zhenhui Lu, Qin Wang, Yan Li.

**Supervision:** Xiangyang Zhu.

**Writing – original draft:** Can Xing.

**Writing – review & editing:** Xiangyang Zhu.
